# Supplementary material for: Brain Microstructure and Obesity Risk in Early Childhood: Insights from Restriction Spectrum Imaging
Source: bioRxiv. 2025 Sep 19:2025.09.19.677172. Preprint. [Version 1] doi: 10.1101/2025.09.19.677172 (PMC12458170; doi:10.1101/2025.09.19.677172)
Supplement: Supplement 1 [file media-1.pdf]

TABLE 1 Associations of anthropometric measures with RNT

Brain Region	β	SE	t	p	95% CI Lower	95% CI Upper	r_{sp}	FDR_p
**Body Mass Index**								
Left Insula	0.15	0.08	1.84	0.07	-0.01	0.31	0.15	0.14
Right Insula	0.27	0.08	3.39	0.00	0.11	0.42	0.27	0.01
Nucleus Accumbens	0.17	0.08	2.18	0.03	0.02	0.33	0.17	0.10
Putamen	0.14	0.08	1.83	0.07	-0.01	0.30	0.15	0.14
Caudate	0.07	0.08	0.86	0.39	-0.09	0.23	0.07	0.49
Pallidum	0.21	0.08	2.71	0.01	0.06	0.36	0.21	0.04
Ventral Diencephalon	-0.02	0.08	-0.25	0.80	-0.17	0.13	-0.02	0.89
Thalamus	0.09	0.08	1.23	0.22	-0.06	0.24	0.10	0.37
Amygdala	-0.00	0.08	-0.02	0.99	-0.16	0.16	-0.00	0.99
Hippocampus	0.08	0.08	0.97	0.34	-0.08	0.24	0.08	0.48
**Waist Circumference**								
Left Insula	0.16	0.09	1.83	0.07	-0.01	0.33	0.15	0.29
Right Insula	0.14	0.09	1.52	0.13	-0.04	0.32	0.14	0.29
Nucleus Accumbens	0.18	0.09	2.03	0.04	0.00	0.36	0.18	0.29
Putamen	0.07	0.09	0.78	0.44	-0.11	0.25	0.07	0.54
Caudate	0.13	0.09	1.36	0.18	-0.06	0.31	0.12	0.29
Pallidum	0.13	0.09	1.48	0.14	-0.04	0.30	0.13	0.29
Ventral Diencephalon	-0.00	0.08	-0.05	0.96	-0.17	0.16	-0.00	0.96
Thalamus	0.11	0.08	1.36	0.17	-0.05	0.27	0.12	0.29
Amygdala	0.06	0.09	0.70	0.49	-0.12	0.25	0.06	0.54
Hippocampus	0.11	0.10	1.14	0.26	-0.08	0.30	0.11	0.37
**% Body Fat**								
Left Insula	0.12	0.09	1.45	0.15	-0.04	0.29	0.12	0.37
Right Insula	0.17	0.08	2.09	0.04	0.01	0.34	0.17	0.22
Nucleus Accumbens	0.12	0.08	1.45	0.15	-0.04	0.29	0.12	0.37
Putamen	0.08	0.09	0.94	0.35	-0.09	0.26	0.09	0.58
Caudate	0.05	0.08	0.56	0.58	-0.12	0.22	0.05	0.58
Pallidum	0.17	0.08	2.03	0.04	0.00	0.33	0.17	0.22
Ventral Diencephalon	-0.04	0.08	-0.56	0.58	-0.20	0.11	-0.05	0.58
Thalamus	0.06	0.08	0.72	0.47	-0.10	0.22	0.06	0.58
Amygdala	-0.10	0.09	-1.13	0.26	-0.27	0.07	-0.10	0.52
Hippocampus	0.05	0.09	0.60	0.55	-0.12	0.22	0.05	0.58
**Categorized as Obese**								
Left Insula	0.04	0.08	0.48	0.63	-0.12	0.20	0.04	0.70
Right Insula	0.12	0.08	1.55	0.12	-0.03	0.28	0.12	0.53
Nucleus Accumbens	0.07	0.08	0.88	0.38	-0.09	0.22	0.07	0.53
Putamen	0.09	0.08	1.12	0.26	-0.07	0.24	0.09	0.53
Caudate	0.01	0.08	0.09	0.93	-0.15	0.17	0.01	0.93
Pallidum	0.11	0.08	1.43	0.15	-0.04	0.26	0.12	0.53
Ventral Diencephalon	0.06	0.08	0.80	0.42	-0.09	0.21	0.07	0.53
Thalamus	0.08	0.07	1.11	0.27	-0.06	0.23	0.09	0.53
Amygdala	0.09	0.08	1.12	0.27	-0.07	0.24	0.09	0.53
Hippocampus	0.07	0.08	0.91	0.36	-0.09	0.23	0.07	0.53

TABLE 2 Associations of anthropometric measures with RNI

Brain Region	β	SE	t	p	95% CI Lower	95% CI Upper	r_{sp}	FDR_p
**Body Mass Index**								
Left Insula	0.17	0.08	2.15	0.03	0.01	0.33	0.17	0.07
Right Insula	0.22	0.08	2.80	0.01	0.07	0.38	0.22	0.03
Nucleus Accumbens	0.22	0.07	2.99	0.00	0.08	0.37	0.24	0.03
Putamen	0.18	0.07	2.68	0.01	0.05	0.31	0.21	0.03
Caudate	0.09	0.08	1.22	0.23	-0.06	0.25	0.10	0.28
Pallidum	0.15	0.07	2.17	0.03	0.01	0.28	0.17	0.07
Ventral Diencephalon	0.03	0.08	0.40	0.69	-0.12	0.18	0.03	0.69
Thalamus	0.13	0.07	1.72	0.09	-0.02	0.27	0.14	0.12
Amygdala	0.09	0.07	1.16	0.25	-0.06	0.23	0.09	0.28
Hippocampus	0.16	0.08	2.00	0.05	0.00	0.31	0.16	0.08
**Waist Circumference**								
Left Insula	0.17	0.09	1.95	0.05	-0.00	0.34	0.16	0.29
Right Insula	0.09	0.09	0.93	0.35	-0.10	0.28	0.09	0.39
Nucleus Accumbens	0.16	0.09	1.87	0.06	-0.01	0.34	0.17	0.29
Putamen	0.10	0.08	1.28	0.20	-0.05	0.25	0.11	0.29
Caudate	0.11	0.10	1.19	0.24	-0.08	0.30	0.12	0.30
Pallidum	0.10	0.08	1.29	0.20	-0.05	0.25	0.11	0.29
Ventral Diencephalon	0.03	0.08	0.41	0.68	-0.13	0.20	0.04	0.68
Thalamus	0.13	0.08	1.63	0.10	-0.03	0.29	0.14	0.29
Amygdala	0.12	0.08	1.46	0.15	-0.04	0.29	0.13	0.29
Hippocampus	0.13	0.09	1.46	0.15	-0.05	0.30	0.13	0.29
**% Body Fat**								
Left Insula	0.13	0.08	1.59	0.11	-0.03	0.30	0.13	0.23
Right Insula	0.15	0.08	1.79	0.08	-0.02	0.31	0.15	0.21
Nucleus Accumbens	0.19	0.08	2.40	0.02	0.03	0.35	0.20	0.18
Putamen	0.16	0.07	2.10	0.04	0.01	0.30	0.18	0.19
Caudate	0.08	0.08	1.02	0.31	-0.08	0.25	0.09	0.39
Pallidum	0.12	0.07	1.73	0.09	-0.02	0.27	0.15	0.21
Ventral Diencephalon	0.04	0.08	0.52	0.60	-0.12	0.20	0.04	0.60
Thalamus	0.10	0.08	1.28	0.20	-0.05	0.25	0.11	0.29
Amygdala	0.05	0.08	0.58	0.57	-0.11	0.20	0.05	0.60
Hippocampus	0.12	0.08	1.41	0.16	-0.05	0.28	0.12	0.27
**Categorized as Obese**								
Left Insula	0.07	0.08	0.88	0.38	-0.09	0.22	0.07	0.38
Right Insula	0.11	0.08	1.45	0.15	-0.04	0.27	0.12	0.21
Nucleus Accumbens	0.15	0.07	2.07	0.04	0.01	0.30	0.17	0.13
Putamen	0.15	0.07	2.29	0.02	0.02	0.28	0.18	0.13
Caudate	0.08	0.08	1.07	0.28	-0.07	0.23	0.09	0.32
Pallidum	0.10	0.07	1.57	0.12	-0.03	0.24	0.13	0.20
Ventral Diencephalon	0.08	0.07	1.06	0.29	-0.07	0.23	0.09	0.32
Thalamus	0.12	0.07	1.71	0.09	-0.02	0.27	0.14	0.18
Amygdala	0.15	0.07	2.15	0.03	0.01	0.29	0.17	0.13
Hippocampus	0.15	0.08	1.90	0.06	-0.01	0.30	0.15	0.15

TABLE 3 Associations of anthropometric measures with RND

Brain Region	β	SE	t	p	95% CI Lower	95% CI Upper	r_{sp}	FDR_p
**Body Mass Index**								
Left Insula	0.13	0.08	1.62	0.11	-0.03	0.30	0.13	0.27
Right Insula	0.28	0.08	3.53	0.00	0.12	0.43	0.28	0.01
Nucleus Accumbens	0.13	0.08	1.63	0.11	-0.03	0.29	0.13	0.27
Putamen	0.08	0.08	0.99	0.32	-0.08	0.24	0.08	0.62
Caudate	0.06	0.08	0.69	0.49	-0.11	0.22	0.06	0.70
Pallidum	0.19	0.08	2.34	0.02	0.03	0.34	0.19	0.10
Ventral Diencephalon	-0.04	0.08	-0.48	0.63	-0.19	0.12	-0.04	0.70
Thalamus	0.07	0.08	0.89	0.37	-0.08	0.22	0.07	0.62
Amygdala	-0.05	0.08	-0.57	0.57	-0.21	0.11	-0.05	0.70
Hippocampus	0.03	0.08	0.34	0.73	-0.13	0.19	0.03	0.73
**Waist Circumference**								
Left Insula	0.15	0.09	1.68	0.09	-0.03	0.32	0.14	0.31
Right Insula	0.16	0.09	1.78	0.08	-0.02	0.34	0.16	0.31
Nucleus Accumbens	0.18	0.09	1.94	0.05	-0.00	0.35	0.17	0.31
Putamen	0.03	0.09	0.36	0.72	-0.15	0.22	0.03	0.82
Caudate	0.13	0.09	1.40	0.16	-0.05	0.31	0.12	0.41
Pallidum	0.12	0.09	1.27	0.21	-0.06	0.30	0.11	0.41
Ventral Diencephalon	-0.02	0.08	-0.22	0.82	-0.19	0.15	-0.02	0.82
Thalamus	0.09	0.08	1.12	0.26	-0.07	0.26	0.10	0.44
Amygdala	0.02	0.09	0.25	0.80	-0.16	0.21	0.02	0.82
Hippocampus	0.09	0.10	0.88	0.38	-0.11	0.29	0.08	0.55
**% Body Fat**								
Left Insula	0.11	0.09	1.33	0.18	-0.06	0.28	0.11	0.46
Right Insula	0.18	0.08	2.17	0.03	0.02	0.34	0.18	0.29
Nucleus Accumbens	0.08	0.09	0.88	0.38	-0.09	0.24	0.07	0.63
Putamen	0.01	0.09	0.13	0.90	-0.17	0.19	0.01	0.92
Caudate	0.02	0.08	0.29	0.77	-0.14	0.19	0.02	0.92
Pallidum	0.15	0.08	1.73	0.09	-0.02	0.32	0.15	0.29
Ventral Diencephalon	-0.07	0.08	-0.88	0.38	-0.23	0.09	-0.07	0.63
Thalamus	0.03	0.08	0.39	0.70	-0.13	0.20	0.03	0.92
Amygdala	-0.16	0.08	-1.88	0.06	-0.33	0.01	-0.16	0.29
Hippocampus	0.01	0.09	0.11	0.92	-0.16	0.18	0.01	0.92
**Categorized as Obese**								
Left Insula	0.02	0.08	0.29	0.78	-0.14	0.18	0.02	0.81
Right Insula	0.12	0.08	1.56	0.12	-0.03	0.28	0.12	0.81
Nucleus Accumbens	0.02	0.08	0.25	0.80	-0.14	0.18	0.02	0.81
Caudate	-0.02	0.08	-0.24	0.81	-0.18	0.14	-0.02	0.81
Pallidum	0.09	0.08	1.11	0.27	-0.07	0.24	0.09	0.81
Ventral Diencephalon	0.05	0.08	0.65	0.51	-0.10	0.20	0.05	0.81
Thalamus	0.06	0.08	0.76	0.45	-0.09	0.21	0.06	0.81
Amygdala	0.04	0.08	0.46	0.65	-0.12	0.20	0.04	0.81
Hippocampus	0.02	0.08	0.30	0.77	-0.13	0.18	0.02	0.81

TABLE 4 Associations of anthropometric measures with HNT

Brain Region	β	SE	t	p	95% CI Lower	95% CI Upper	r_{sp}	FDR_p
**Body Mass Index**								
Left Insula	-0.02	0.08	-0.18	0.85	-0.18	0.15	-0.01	0.91
Right Insula	-0.22	0.08	-2.66	0.01	-0.38	-0.06	-0.21	0.09
Nucleus Accumbens	-0.04	0.08	-0.45	0.66	-0.20	0.13	-0.04	0.91
Putamen	-0.10	0.08	-1.22	0.23	-0.26	0.06	-0.10	0.63
Caudate	-0.01	0.08	-0.12	0.91	-0.17	0.15	-0.01	0.91
Pallidum	-0.18	0.08	-2.26	0.03	-0.33	-0.02	-0.18	0.13
Ventral Diencephalon	0.02	0.07	0.26	0.80	-0.13	0.17	0.02	0.91
Thalamus	-0.04	0.08	-0.51	0.61	-0.19	0.12	-0.04	0.91
Amygdala	0.09	0.08	1.15	0.25	-0.07	0.25	0.09	0.63
Hippocampus	0.01	0.08	0.15	0.88	-0.15	0.18	0.01	0.91
**Waist Circumference**								
Left Insula	-0.05	0.09	-0.59	0.56	-0.22	0.12	-0.05	0.71
Right Insula	-0.15	0.09	-1.70	0.09	-0.32	0.02	-0.14	0.46
Nucleus Accumbens	-0.07	0.09	-0.80	0.43	-0.25	0.10	-0.07	0.71
Putamen	-0.05	0.09	-0.57	0.57	-0.22	0.12	-0.05	0.71
Caudate	-0.02	0.09	-0.28	0.78	-0.19	0.15	-0.02	0.78
Pallidum	-0.10	0.09	-1.12	0.27	-0.28	0.08	-0.10	0.71
Ventral Diencephalon	0.06	0.08	0.71	0.48	-0.10	0.21	0.06	0.71
Thalamus	-0.03	0.08	-0.38	0.70	-0.19	0.13	-0.03	0.78
Amygdala	0.16	0.09	1.81	0.07	-0.02	0.34	0.16	0.46
Hippocampus	0.07	0.09	0.78	0.44	-0.11	0.25	0.07	0.71
**% Body Fat**								
Left Insula	-0.06	0.08	-0.73	0.47	-0.23	0.10	-0.06	0.78
Right Insula	-0.21	0.08	-2.53	0.01	-0.38	-0.05	-0.21	0.12
Nucleus Accumbens	-0.06	0.08	-0.74	0.46	-0.23	0.10	-0.06	0.78
Putamen	-0.08	0.09	-0.90	0.37	-0.24	0.09	-0.07	0.78
Caudate	-0.01	0.08	-0.06	0.95	-0.17	0.16	-0.01	0.95
Pallidum	-0.15	0.08	-1.77	0.08	-0.31	0.02	-0.15	0.39
Ventral Diencephalon	0.02	0.08	0.20	0.84	-0.14	0.17	0.02	0.94
Thalamus	-0.04	0.08	-0.51	0.61	-0.20	0.12	-0.04	0.87
Amygdala	0.03	0.09	0.31	0.76	-0.15	0.21	0.03	0.94
Hippocampus	-0.11	0.09	-1.20	0.23	-0.29	0.07	-0.11	0.78
**Categorized as Obese**								
Left Insula	0.02	0.08	0.20	0.84	-0.14	0.18	0.02	1.00
Right Insula	-0.17	0.08	-2.17	0.03	-0.33	-0.02	-0.17	0.16
Nucleus Accumbens	-0.01	0.08	-0.09	0.93	-0.17	0.15	-0.01	1.00
Putamen	-0.04	0.08	-0.46	0.65	-0.19	0.12	-0.04	0.92
Caudate	0.05	0.08	0.57	0.57	-0.11	0.20	0.05	0.92
Pallidum	-0.07	0.08	-0.94	0.35	-0.23	0.08	-0.08	0.92
Ventral Diencephalon	0.04	0.07	0.56	0.58	-0.10	0.19	0.04	0.92
Thalamus	-0.00	0.08	-0.01	1.00	-0.15	0.15	-0.00	1.00
Amygdala	0.17	0.08	2.17	0.03	0.02	0.33	0.17	0.16
Hippocampus	-0.06	0.08	-0.70	0.48	-0.22	0.10	-0.06	0.92
